# Comparative Analysis of Volatile Defensive Secretions of Three Species of Pyrrhocoridae (Insecta: Heteroptera) by Gas Chromatography-Mass Spectrometric Method

**DOI:** 10.1371/journal.pone.0168827

**Published:** 2016-12-20

**Authors:** Jan Krajicek, Martina Havlikova, Miroslava Bursova, Martin Ston, Radomir Cabala, Alice Exnerova, Pavel Stys, Zuzana Bosakova

**Affiliations:** 1 Department of Analytical Chemistry, Faculty of Science, Charles University in Prague, Prague, Czech Republic; 2 Toxicology Department, Institute of Forensic Medicine and Toxicology, General University Hospital in Prague, Prague, Czech Republic; 3 Department of Zoology, Faculty of Science, Charles University in Prague, Prague, Czech Republic; USDA-ARS Beltsville Agricultural Research Center, UNITED STATES

## Abstract

The true bugs (Hemiptera: Heteroptera) have evolved a system of well-developed scent glands that produce diverse and frequently strongly odorous compounds that act mainly as chemical protection against predators. A new method of non-lethal sampling with subsequent separation using gas chromatography with mass spectrometric detection was proposed for analysis of these volatile defensive secretions. Separation was performed on Rtx-200 column containing fluorinated polysiloxane stationary phase. Various mechanical irritation methods (ultrasonics, shaking, pressing bugs with plunger of syringe) were tested for secretion sampling with a special focus on non-lethal irritation. The preconcentration step was performed by sorption on solid phase microextraction (SPME) fibers with different polarity. For optimization of sampling procedure, *Pyrrhocoris apterus* was selected. The entire multi-parameter optimization procedure of secretion sampling was performed using response surface methodology. The irritation of bugs by pressing them with a plunger of syringe was shown to be the most suitable. The developed method was applied to analysis of secretions produced by adult males and females of *Pyrrhocoris apterus*, *Pyrrhocoris tibialis* and *Scantius aegyptius* (all Heteroptera: Pyrrhocoridae). The chemical composition of secretion, particularly that of alcohols, aldehydes and esters, is species-specific in all three pyrrhocorid species studied. The sexual dimorphism in occurrence of particular compounds is largely limited to alcohols and suggests their epigamic intraspecific function. The phenetic overall similarities in composition of secretion do not reflect either relationship of species or similarities in antipredatory color pattern. The similarities of secretions may be linked with antipredatory strategies. The proposed method requires only a few individuals which remain alive after the procedure. Thus secretions of a number of species including even the rare ones can be analyzed and broadly conceived comparative studies can be carried out.

## Introduction

Chemical intraspecific and interspecific communication is essential for most animals, and the low-molecular organic compounds participate in regulation of behavior, synchronization of physiological and developmental activities, and act as secondary defenses against predators, parasitoids, and parasites. The organic compounds functioning in interorganismal communication are called infochemicals [1]. These compounds also take part in aposematic signaling and advertise the prey noxiousness to the potential predators by means of olfactory or gustatory cues [2, 3]. These chemical warning signals are often combined with visual and vibrational (acoustic) signals or cues in a multimodal antipredatory signaling system [4].

The true bugs (Hemiptera: Heteroptera) is a large, diversified and cosmopolitan group of sucking insects including 95 families and over 50,000 described species [5]. They inhabit all the major biomes, from terrestrial to fresh-water and marine, and include such well known families as water scorpions, backswimmers, water striders, assassin bugs, plant bugs, bed bugs and shield bugs. Many species are phytophagous and true bugs are ranked as the fourth major insect group of agricultural pests [6]. Some species are carnivorous feeding on arthropods, gastropods and even small aquatic vertebrates. The predatory true bugs may function in regulation of arthropod pests of cultivated plants while the hematophagous species may be vectors of human and animal diseases [7].

Nearly all true bugs (Insecta: Hemiptera: Heteroptera) are chemically defended against predators and produce defense infochemicals which are either autogenous (synthesized by bugs themselves from precursors contained in food; [6]) or sequestered (received in food non-metabolized and stored in special reservoirs; [7]). The glands producing defense secretion and their reservoirs differ between the larvae (nymphs) and adults [8]. The larvae produce defense secretion from larval dorsoabdominal glands (DAGs). The adult Heteroptera have lost DAGs or retained and transformed them to produce pheromones and allelochemicals, but not infochemicals with defensive function. Nearly all adult Heteroptera produce defense secretion from methathoracic glands (MTGs, scent glands). In some true bug taxa there are additional specialized prothoracic and mainly abdominal glands producing a variety of infochemicals, some of purely defensive function [9–14]. The identity of the antipredatory chemicals and the composition of secretion may be taxon-specific and sex-dependent [1]. The antipredatory infochemicals of true bugs are mostly volatile and strongly odorous, often repellent for vertebrate predators [15], and may be toxic for predatory arthropods and even for the secreting bug itself [16]. Short-chain alcohols, aldehydes, oxo-aldehydes, ketones, esters, alkanes, organic acids, monoterpenes and aromatic alcohol/aldehydes are typical compounds, and the composition of the secretion may depend on the physiological, nutritional and developmental state of the individual and on the season [17].

Most adult terrestrial Heteroptera release their defensive secretions readily upon attack. However, some true bugs (e.g. the firebugs, family Pyrrhocoridae) are reluctant to release their secretion because of behavioral constraints, reduced metathoracic glands (MTGs) or anatomical architecture of their secretion releasing MTGs system [18]. Even when not released, the secretion may still be effective by making a predator sick after consuming the bug, and this experience may induce learned aversion [15, 19].

Several procedures have been proposed for sampling volatile secretions; most of them require killing the insects. One of these procedures is rinsing the bug with dichloromethane, which kills it in a few seconds [20]. This method has the disadvantage that the sample is greatly diluted and substances that are not derived from the secretion but, e.g., from the surface of the body, can also be extracted. Another method is anaesthetizing the bugs using CO_2_ or ethylacetate or killing them by freezing with subsequent dissection of the glands and extraction of their content with methyl-*tert*-butylether, hexane or dichlormethane [11, 21–28], or puncturing the gland with a glass capillary [29]. Such procedures are appropriate for studies of large species (e.g. shield bugs, Pentatomidae) in which only a few individuals are needed for dissection and extraction of sufficient quantities of secretion [14, 25, 30]. On the other hand, the method is overly time-consuming in smaller species in which it is necessary to dissect a large number of individuals (Farine et al. used up to 2000 specimens of *Pyrrhocoris apterus* [16]). Non-lethal sampling methods include blowing air across a vessel containing the bugs with subsequent collection of the substances on various types of adsorbents, such as Super Q, active carbon or Porapaq Q [31–34], sorption on a fiber (SPME) [35–37] or placing a paper napkin between the bugs and subsequent extraction of the napkin with dichloromethane [38]. The relatively newest procedure involves a temperature-desorption system that was used for analyzing pheromones [39, 40]. However, these procedures do not include any irritation of the insects that would simulate a predator attack and assure the discharging of the defensive secretion.

Gas chromatography (GC) combined with flame ionization, mass spectrometric or electro-antennographic detection has been used for analysis of the secretions of true bugs [23, 25, 37, 41–48]. Mainly non-polar DB- or HB-5 capillary columns or parallel connected non-polar DB-1 and polar DB-WAX capillary columns have been used [20, 28, 36, 39, 41]. Because the secretions contain many different substances, preliminary separation can be carried out using high performance liquid chromatography prior to gas chromatography [49], but recently comprehensive two-dimensional gas chromatography (GCxGC) has begun to be used [24, 50]. Substances are identified by comparison with a mass spectrometric database and by nuclear magnetic resonance [33].

Because of the great complexity of the secretion samples and the number of parameters that can affect the sampling process, it can be beneficious to employ the process optimization procedures that combine all the tested parameters together at the same time and substantially reduce the number of analyses required. Response surface methodology (RSM) is based on the fit of a polynomial equation to the multidimensional (multiparametric) experimental data which describes the behavior of the data set with the objective of making statistical predictions. RSM is a general approach for designing experiments; it efficiently reduces the development time and costs of the method. The main advantage of RSM as compared with the currently most widely used approach to optimization, namely one-factor-at-a-time OFAT (one parameter is changed while the others are fixed during optimization) is that only the most important experimental parameters are selected and optimized and that more than one parameter are changed simultaneously in one experiment according to the computer-designed plan of experiments [51–53].

In this study three species of the family Pyrrhocoridae, namely the Palaearctic *Pyrrhocoris apterus* (L.), Oriental *P*. *tibialis* (Stål), and Mediterranean *Scantius aegyptius* (L.) were chosen to analyze and compare their secretions since they are closely related but differ in many biologically important traits. *Pyrrhocoris apterus* and *P*. *tibialis* are congeneric, closely related but largely allopatric species [54]. They have similar host plant (various Malvaceae incl. *Tilia* spp.; in case of *P*. *apterus* also an introduced locust tree, *Robinia pseudacacia*) but a different life style, and both are reluctant to discharge their defensive secretion upon an attack. *P*. *apterus* is gregarious, mostly brachypterous, flightless species, and possesses a warning coloration of conspicuous red-and-black color pattern. *P*. *tibialis* is non-gregarious, readily flying, and rather cryptically colored in shades of gray and brown. *Scantius aegyptius* is a macropterous (but flightless), epigeic, Mediterranean species occupying the southwestern part of the range of *P*. *apterus* and sharing with the latter not only similar red-and-black warning coloration, but also the same habitat and major hostplant (*Malva neglecta*; Malvaceae). Like both *Pyrrhocoris* species, it is also reluctant to discharge its defensive secretion.

This study was carried out to i) develop a non-lethal method for sampling the defensive secretion of true bugs using various kinds of mechanical irritation that would simulate the attack of a predator, and also find a method suitable for the species that are reluctant to release their secretion; ii) select the most advantageous sampling of the secretion using SPME fibers with various polarities for subsequent GC-MS analysis; iii) optimize the sampling using the response surface methodology; iv) employ the developed method for analysis and comparison of infochemicals secreted by adults of both sexes in the three species of Pyrrhocoridae differing in their biology and antipredatory strategies, and to assess the similarities and differences in secretions.

MTG secretion of *P*. *apterus* has already been analyzed by Farine [20] using dissected MTGs and their reservoirs. Except for *P*. *apterus*, *Dysdercus intermedius* Distant, *D*. *superstitiosus* (F.), *D*. *fasciatus* Signoret, and *D*. *cingulatus* (F.) defensive secretion of no other pyrrhocorid species have been analyzed [20], and to our best knowledge such comparisons of secretion in closely related species of Heteroptera are surprisingly rare.

## Materials and Methods

### Chemicals

A mixture of *n*-alkanes (C_8_—C_20_) dissolved in hexane for retention index determination was purchased from Fluka (Buchs, Switzerland). Reference compounds, (*E*)-2-hexen-1-ol (> 96%), (*E*)-2-octen-1-ol (> 97%), 1-decyne (> 98%), 1-dodecene (≥ 99%), (*E*,*E*)-2,4-hexadienal (≥ 97%), 2-cyclohexen-1-ol (> 95%), (*E*)-2-hexenal (≥ 99%), (*E*)-2-octenal (≥ 95%), 4-*tert*-butylcyclohexyl acetate (mixture of *E* and *Z*, > 98%), allyl cyclohexanepropionate (≥ 98%), cyclopentanemethanol (≥ 98%), cyclopentanol (≥ 99%), cyclopentanone (≥ 99%), decanal (≥ 98%), 1-dodecanol (≥ 98%), dodecyl acetate (> 97%), hexyl acetate (≥ 99%), hexyl salicylate (≥ 99%), limonene (> 97%), nonanal (≥ 97%), octyl acetate (≥ 99%), *p*-cymene (> 99%), 1-tridecanol (≥ 99%), and α-hexylcinnamaldehyde (≥ 95%) were supplied by Sigma Aldrich (Munich, Germany).

### Instrumentation, separation conditions and identification of the volatile components

The analyses were performed using a GCMS-QP2010 Plus instrument (Shimadzu, Japan), equipped with 20 m × 0.15 mm i.d., 0.15 μm film thickness Rtx-200 column (trifluoropropylmethyl polysiloxane stationary phase, Restek, USA). This stationary phase was selected as a general purpose one with respect to its unique ability to separate substances in a relatively broad polarity range. Helium (99.999%, Linde, Czech Republic) was used as the carrier gas at a constant linear flow rate of 35 cm s^-1^. Splitless-mode injection with SPME liner at 250°C was employed (split valve closed for 1 min). The oven temperature was maintained at 35°C for 3 min, ramped at 5°C min^-1^ to 130°C, then ramped at 20°C min^-1^ to 300°C and then maintained for 5 min (total run time, 35.50 min). The mass spectrometer was operated in the scan mode (m/z 35—500). The ion source and interface temperatures were 200 and 250°C, respectively.

The data were collected and evaluated using the GCMS software (Shimadzu, Japan), Origin 8 (Origin Lab corporation, Northampton, MA, USA), Microsoft Excel 2003 (Microsoft Corporation, Redmond, DC, USA) and Minitab 16 (Minitab Inc., State College, PA, USA) programs. Identification of the secretion components was made by comparing the obtained spectra with those in the NIST 2008 Mass Spectra Library. Series of *n*-alkanes (C8—C20) and reference compounds (see Section *Chemicals*) were analyzed under the same experimental conditions as those used for the samples to either establish the retention indices or confirm the identity of the analytes. Retention times of reference compounds were measured by injection of their diluted solutions in pentane. The dilution was selected for each standard individually in such a way that its retention time was constant and reproducible and did not change with higher dilution. The identity confirmation of the separated compounds was performed by comparing the experimental retention times, retention indices and mass spectra of the compounds with those of concurrently analyzed reference standards.

### Sample preparation and bug samples

For the study of sampling procedure *Pyrrhocoris apterus* was selected, because it is easily available in large numbers [19], it was frequently used as a model in experimental studies [55] and its secretion has already been studied [20]. Moreover, *P*. *apterus* is characterized by a moderate chemical defense with a delayed effect on predator and only releases its defense secretion when it is strongly irritated [15, 19]. This species was used for optimization experiments focused on finding the most efficient approach for extracting and analyzing defensive secretion in Pyrrhocoridae. To optimize sampling conditions for all possible compounds four individuals of *P*. *apterus* were used together, always two males and two females.

Under the optimized sampling and separation conditions the defensive secretions of adults of three closely related true bug species (Hemiptera: Heteroptera: Pyrrhocoridae, the firebugs), were studied: (1) *Pyrrhocoris apterus* (L., 1758) from Prague, Czech Republic, a common Eurasian aposematically colored species introduced to other continents as well, (2) *P*. *tibialis* (Stål, 1874), from Tianjin, China, an East Palaearctic species, and (3) *Scantius aegyptius* (L., 1758) from Greece (Kos Island and Crete), a Mediterranean species. The adults were sexed, fed with seeds of their original host plants (*Tilia cordata* in *P*. *apteru*s, *Alcea rosea* in *S*. *aegyptius*, and *Hibiscus rosa-sinensis* in *P*. *tibialis*), and maintained separately in plastic containers at 25 ± 2°C and photoperiod L16:D8. Three males and three females of each species were used for the individual analysis, the sexes were sampled separately and the analysis was performed in triplicate (*n* = 3).

Three individuals were placed in 4 mL glass vial closed with a polypropylene stopper (Supelco, Bellefonte, PA, USA) with a teflon septum (Supelco, Bellefonte, PA, USA), and the vial was then placed in an ultrasonic bath (Elma, Singen, Germany) or shaker (Heidolph, Schwabach, Germany). In the experiment employing irritation with a plunger, three individuals were placed in a syringe with a barrel volume of 12.5 mL (Eppendorf, Hamburg, Germany), which was placed in an incubated shaker for tempering the specimens (bioSan, Riga, Lithuania). Following a certain tempering time at the given temperature, the bugs were carefully compressed with the plunger of a syringe so that they could not move and the syringe tip was then closed with a rubber stopper. The bugs were compressed with the plunger until a thin liquid film appeared on their body. Then the rubber stopper was removed from the syringe tip, 5 mL of air was drawn in and an SPME (solid phase microextraction) fiber was immediately inserted into the tip of the barrel.

The following experimental conditions were studied for each sampling method: i) irritation with ultrasonics: type of fiber, irritation temperature, intensity of the ultrasonics, irritation time, SPME sampling time and temperature of SPME sorption; ii) for irritation in a shaker: type of fiber, irritation temperature, rate of shaking, irritation time, SPME sampling time and temperature of SPME sorption and iii) for irritation using compression in the barrel of a syringe: type of fiber, temperature prior to compression, tempering time prior to compression, time and temperature of SPME sorption.

The volatile compounds were extracted from the syringe barrel using a manual SPME sampler with a selected fiber assembly (all Supelco, Bellefonte, PA, USA) coated with: i) 7 μm polydimethylsiloxane (PDMS), ii) 85 μm polyacrylate (PA) and iii) triple phase 50/30 μm divinylbenzene/carboxen/polydimethylsiloxane (DVB/CAR/PDMS). Each SPME fiber was thoroughly conditioned for 15 min before and after each analysis in an external syringe oven at 250°C under vacuum. Before each analysis of defensive secretion, a control analysis of SPME sampler itself and SPME sampling of the syringe itself (blank) was performed. The insects were tempered during the sorption on SPME fiber to increase vapor pressure.

### Comparison of secretions between true-bug species

In order to consider the intra- and inter-specific chemical diversity of defensive chemicals, we constructed a cluster tree based on qualitative similarities in chemical profiles among all analysed sampled. The clustering was based on distance matrix, in which the distance between each pair of samples was expressed as a number of compounds exclusively present in one of the samples and absent in the other. Single linkage clustering algorithm was used for tree construction. All 135 detected compounds were considered, including the fully identified compounds as well as partially identified chemicals, characterized by their retention behaviour and mass spectra with similarity (match factor in NIST 2008 data base) lower than 85%. Three independent biological replicates for each species and sex were used, each consisting of a pooled sample of three individuals, except for *P*. *apterus* females, in which only two replicates were available.

## Results and Discussion

### Selection of parameters and response definition

The studied parameters were selected with respect to several factors. One of the main requirements on sampling of volatile secretions was that the bugs would not be killed and destroyed, so the method would be suitable for a limited material of rare species. We found that the critical sampling temperature for *P*. *apterus* is 40°C, because higher temperature is lethal. Therefore, this temperature has been arbitrarily set as the highest applied temperature also for other two tested species. For SPME, the choice of the polarity of the sampling fiber is the most critical condition for reliable and reproducible analysis of a sample. As the detailed composition of the secretions of the studied species of Pyrrhocoridae is unknown and can depend on many factors (described in the introduction), three different SPME fibers with different polarities were studied—polar PA, nonpolar PDMS fibers and composite bipolar DVB/CAR/PDMS fiber covering a wide range of volatilities and polarities. The type of fiber, irritation temperature and time, and the temperature of SPME sorption constituted the basic set of parameters for each of the proposed irritation methods. Ultrasonics was supplemented by the parameter of the ultrasonics intensity and the shaker by the shaking rate parameter. Selected parameters together with their tested levels (low, middle and high) are summarized in Table 1. The individual parameters for sampling using a syringe are listed on Table 2. For this sampling method the parameter of the type of fiber is omitted, because optimization was performed for each fiber separately. Sets of measurements, combining the factors and their levels are given in the Supporting Information (S1–S3 Tables).

**Table 1:** Selected Parameters and Their Tested Levels for Sampling Secretion Using Ultrasonics and Shaker.

Parameter	Level		
	Low	Middle	High
irritation time (min)	1	3	5
irritation temperature (°C)	25	32.5	40
intensity of the ultrasonics (%)	30	65	100
rate of shaking (rpm)	300	750	1200
temperature of SPME sorption (°C)	25	32.5	40
SPME sampling time (min)	30	60	90

**Table 2:** Selected Parameters and Their Tested Levels for Sampling Secretion Using Compression in the Plunger of a Syringe.

Parameter	Level		
	Low	Middle	High
temperature prior to compression (°C)	25	32.5	40
tempering time prior to compression (min)	1	3	5
temperature of SPME sorption (°C)	25	32.5	40
time of SPME sorption (min)	30	60	90

In addition to developing a non-lethal method of sampling volatile secretions, we also attempted to obtain the largest possible amount of secretions. The following responses were chosen: the number of peaks with an area of at least 20,000 arbitrary units (a.u.) and the sum of their areas.

### Optimization procedure

The secretion sampling method was optimized and Tables 3 and 4 provide the obtained optimum parameters.

**Table 3:** Optimum Conditions for the Method of Sampling Secretions Using Ultrasonics and a Shaker.

Parameter	Ultrasonics	Shaker
type of fiber	DVB/CAR/PDMS	DVB/CAR/PDMS
irritation time (min)	1	5
irritation temperature (°C)	25	25
temperature of SPME sorption (°C)	40	40
SPME sampling time (min)	90	30
intensity of the ultrasonics (%)	100	−
rate of shaking (rpm)	−	300
number of peaks	23	15
sum of absolute peaks areas	49 660 000	13 770 000

**Table 4:** Optimum Conditions for the Method of Sampling Secretions Using Compression in the Plunger of a Syringe.

type of fiber	PDMS	PA	DVB/CAR/PDMS
temperature prior to compression (°C)	25	40	**40**
tempering time prior to compression (min)	1	5	**1**
temperature of SPME sorption (°C)	25	40	**40**
time of SPME sorption (min)	30	90	**90**
number of peaks	10	8	**59**
sum of absolute peaks areas	1 092 000	7 079 000	**35 620 000**

It can be seen from Table 3 that, on the basis of the required responses (maximum number of peaks, maximum sum of the areas of all the peaks), the DVB/CAR/PDMS fiber seemed to be the best of the fibers studied. Irritation by ultrasonics was more effective than irritation in a shaker, because it generated a greater number of peaks and maximum sum of the areas of all the peaks. When the secretion was sampled using compression of the bugs by the plunger of a syringe (see Table 4), polar (PA) and nonpolar (PDMS) fibers exhibited much lower responses and again unambiguously the best results were obtained with DVB/CAR/PDMS fibers. The advantageousness of irritation by ultrasonics and compression by the plunger can be seen from comparison of the required responses obtained in all the tested systems. Irritation by ultrasonics generates a smaller number of peaks than compression with a plunger, but the overall response is somewhat higher than when using a plunger. The sampling method employing compression with the plunger of a syringe combined with DVB/CAR/PDMS was chosen as the most advantageous, generating the greatest number of peaks and probably simulating best the real danger to the bug when being seized by a large insectivorous predator, and when releasing its full set of antipredatory chemicals.

**Fig 1:**
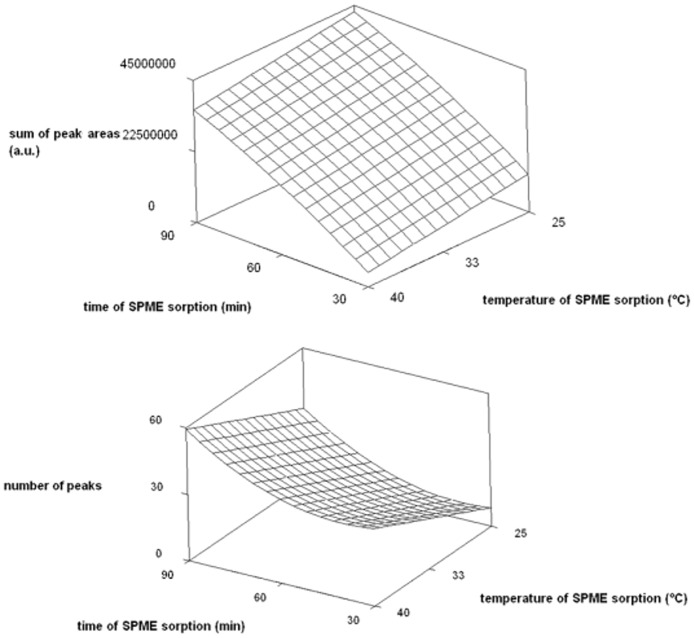
The response surface plot. The plot depicts the dependence of the sum of all the peaks (upper) (a.u., arbitrary units) and the number of peaks (bottom) on the temperature of SPME sorption and time of SPME sorption; irritation by compressing the true bugs with the plunger of a syringe; SPME sorption on a DVB/CAR/PDMS fiber; tempering time prior to compression 1 min; temperature prior to compression 40°C.

For the responses “maximum number of peaks” and “maximum sum of their areas” the coefficients of determination (*R*^2^) were 0.9710 and 0.8720, respectively. These results indicate very good agreement (97.1 and 87.2% of explained variability) between the experimental data and the built up model. Examples of two response surfaces of plots for this sampling method are given in Fig 1, depicting the interrelationships of selected parameters with the response. It can be seen from the two graphs that increasing time of SPME sorption are accompanied by an increase in the areas of all the peaks and also in their number. The increasing temperature of SPME sorption generates decreasing sum of peaks areas but increasing number of peaks which is more important for qualitative analysis.

#### Analysis of defensive secretions in adults of Pyrrhocoris apterus, Pyrrhocoris tibialis and Scantius aegyptius by GC-MS

Under the optimized conditions defensive secretions of all the three studied species were analyzed. Sampling was performed under the conditions listed in Table 4 (bold column) by sorption on a DVB/CAR/PDMS fiber after compressing the bugs with the plunger of a syringe. The actual GC analysis was performed under the conditions described in Section *Sample preparation and bug samples*. The bugs were sexed and the identical experiments were carried out separately for males and females with three individuals in each analysis. Identifications of the individual peaks obtained for males and females of all three studied species, the corresponding retention times, their standard deviations and relative peak areas (*n* = 3) are summarized in Table 5, and only peaks with response (*A*) greater than 20,000 a.u. and similarity (match factor in NIST 08 database) higher than 85% were selected for the identification and following normalization.

**Table 5:** Retention Time, Standard Deviation of Retention Time, Relative Peak Abundance, Similarity of Mass Spectra, and Identification Method of Compounds Identified in the Defensive Secretions of Males and Females of *P*. *apterus*, *P*. *tibialis* and *S*. *aegyptius*.

	Compound	*t*_ret_ (min) ± SD	Relative Abundance (%) and Spectra Similarity (*S*)												Identification
			*Pyrrhocoris apterus*				*Pyrrhocoris tibialis*				*Scantius aegyptius*				
			Male	S	Female	S	Male	S	Female	S	Male	S	Female	S	
**Hydrocarbons**															
1	octan-1,3-diene	3.31 ± 0.06					0.03	87	1.91	95					B
2	3,7-dimethyl-1,6-octadiene	5.38 ± 0.03									0.25	85	0.34	85	B
3	1-decyne	8.05 ± 0.05					4.20	95	0.55	94	0.46	91	2.24	96	A, B
*4*	*p*-cymene	8.54 ± 0.02					0.38	87	0.21	90	0.18	89	0.59	80	A, B
5	undecane	9.16 ± 0.03	0.11	86	0.09	85	1.01	86	0.70	93	0.34	92	0.13	90	A, B
6	6-dodecene	11.79 ± 0.06					0.11	85	0.15	89					B
7	dodecane	11.86 ± 0.07	1.47	96	0.62	96	3.83	97	2.82	98	10.84	97	2.92	97	A, B
8	1-dodecene	11.97± 0.03					1.19	90	1.42	95	2.89	93	1.13	95	A, B
9	tridecane	14.45 ± 0.05	10.85	96	10.29	96	1.29	92	1.16	96	0.63	92	0.90	90	A, B
10	tetradecane	16.91 ± 0.02	1.54	95	0.26	94	17.72	97	6.62	97	7.62	97	9.01	95	A, B
11	pentadecane	19.52 ± 0.06	0.55	85	0.48	87	3.05	87	2.14	89	2.08	92	2.54	89	A, B
12	hexadecane	21.41 ± 0.07	0.25	90	0.26	89	4.20	96	2.50	96	1.95	94	2.42	92	A, B
**Alcohols**															
13	cyclopentanol	3.55 ± 0.03									0.28	87	0.14	86	A, B
14	2,3-butanediol	5.02 ± 0.06	1.72	96	4.12	93									B
15	(*E*)-2-hexen-1-ol	5.59 ± 0.03							15.06	96					A, B
16	cyclopentanemethanol	6.42 ± 0.05									11.42	96	12.96	94	A, B
17	2-cyclohexen-1-ol	6.88 ± 0.05									12.07	85	18.85	88	A, B
18	2-propyl-1-pentanol	9.70 ± 0.02			3.46	86									B
19	(*E*)-2-octen-1-ol	11.16 ± 0.06							9.65	96					A, B
20	2,6-dimethyl-7-octen-2-ol	11.60 ± 0.08					1.67	93	1.17	94	1.75	95	0.62	89	B
21	2-methoxyphenol	13.52 ± 0.05											1.25	85	B
22	isotridecanol	15.42 ± 0.07									1.47	88	1.64	89	B
23	2-isopropyl-5-methyl-1-heptanol	15.61 ± 0.05					2.47	88	1.38	88					B
24	2-methyl-1-undecanol	20.22 ± 0.05					0.18	75	0.14	86					B
25	3,7,11-trimethyl-1-dodecanol	20.74 ± 0.04									1.23	87	0.50	85	B
26	1-dodecanol	21.10 ± 0.02	0.14	93	0.10	91	9.48	98	7.52	98	9.21	97	10.01	96	A, B
27	3,5-bis(1,1-dimethylethyl)phenol	21.22 ± 0.06					2.81	85	1.10	87	1.58	86	1.87	85	B
28	2-ethyl-1-dodecanol	22.27 ± 0.04					0.30	86	0.06	90					B
29	1-tridecanol	22.99 ± 0.04					1.08	90	0.79	95	0.81	94	0.95	90	A, B
**Aldehydes**															
30	(*Z*)-3-hexenal	5.23 ± 0.09	0.37	85	0.52	85									B
31	hexanal	5.56 ± 0.05	0.09	88	0.09	90	0.17	89	0.53	86					B
32	(*E*)-2-hexenal	8.17 ± 0.04	18.52	97	23.81	97	0.38	86	3.28	96					A, B
33	(*E*,*E*)-2,4-hexadienal	10.11 ± 0.07	1.07	87	0.43	85			0.12	88					A, B
34	nonanal	13.96 ± 0.06	6.20	97	1.83	97	0.92	91	1.01	92	0.49	90	0.78	96	A, B
35	(*E*)-2-octenal	14.06 ± 0.06	1.55	87	2.56	85	0.64	86	2.95	95					A, B
36	decanal	16.57 ± 0.05	1.64	95	1.05	98	0.68	89	0.53	89	0.74	91	1.21	90	A, B
37	tetradecanal	21.36 ± 0.08							0.38	88					B
38	α-hexylcinnamaldehyde	25.55 ± 0.03					0.98	87	0.69	92	1.12	94	0.66	92	A, B
**Ketones**															
39	Cyclopentanone	6.27 ± 0.05									3.77	97	0.34	85	A, B
40	2-methyl-2-cyclopenten-1-one	8.48 ± 0.06									1.22	85	2.36	85	B
41	2-ethylcyclohexanone	18.55 ± 0.06					0.62	89	0.36	81					B
42	1-(4-*tert*-butylphenyl)propan-2-one	23.13 ± 0.04					0.54	89	0.15	86					B
**Esters**															
43	1-butanol-3-methyl acetate	7.18 ± 0.06									0.55	91	0.40	89	B
44	2-buten-1-ol-3-methyl acetate	8.19 ± 0.03									0.28	85	0.23	88	B
45	hexyl acetate	10.12 ± 0.05									0.44	91	1.32	90	A, B
46	methyl 2-hydroxy-3-methyl pentanoate	10.25 ± 0.06	14.01	87	4.60	87									B
47	(*E*)-2-hexen-1-ol acetate	10.79 ± 0.07							0.47	94	0.63	95	0.87	94	B
48	acetic acid, undec-2-enyl ester	15.92 ± 0.05									1.19	88	0.62	89	B
49	octyl acetate	16.14 ± 0.04									0.75	91	0.37	90	A, B
50	4-*tert*-butylcyclohexyl acetate ^a^	18.64 ± 0.02					3.91	90	3.24	90	4.37	91	3.90	92	A, B
51	4-*tert*-butylcyclohexyl acetate ^a^	19.65 ± 0.06					0.72	88	0.46	87	0.18	85	0.58	86	A, B
52	allyl cyclohexane propionate	20.93 ± 0.06					1.25	88	1.68	87	1.32	86	0.79	87	A, B
53	indan-1,3-diol monoacetate	21.59 ± 0.03					2.23	89	1.38	89					B
54	verdyl acetate	21.64 ± 0.07					1.28	86	1.01	87					B
55	phenylethyl isovalerate	22.84 ± 0.06	0.53	95	0.05	90									B
56	isopropyl laurate	23.89 ± 0.06	0.21	87	0.33	85									B
57	dodecyl acetate	23.95 ± 0.04					0.64	85	0.41	91	0.38	91	0.35	90	A, B
58	hexyl salicylate	24.87 ± 0.05					0.27	87	0.39	92	0.05	94	0.25	92	A, B
59	methyl dihydrojasmonate	25.59 ± 0.06	0.23	86	2.22	85									B
60	2-ethylhexyl salicylate	25.68 ± 0.05	0.21	89	2.51	88									B
**Others**															
61	3-methylbutanoic acid	6.40 ± 0.03	0.91	91	0.15	94	1.70	96	1.81	97					B
62	limonene	7.65 ± 0.07					7.38	93	5.09	93	3.93	93	3.67	91	A, B
63	(*E*)-2-hexenoic acid	11.62 ± 0.07	0.11	86	0.15	92									B
64	2-ethylhexanoic acid	13.28 ± 0.04	1.20	86											B
65	1-ethoxynaphtalene	23.27 ± 0.06					0.83	85	0.47	88	0.35	90	0.45	92	B
66	1-methoxyoctane	25.30 ± 0.06	1.08	85	4.13	88									B

^a^ *E* or *Z* isomer

*t*_ret_ is the retention time of the relevant substance, SD standard deviation (*n* = 3), relative abundances (% areas of the relevant peaks) as a result of chromatogram internal normalization. The methods used for the identification: A—retention time and mass spectrum of the relevant substance was compared with the reference compound; B—the mass spectrum of the relevant substance was compared with NIST 2008 mass spectra library; S—similarity of the compound spectrum with the spectrum in the NIST 2008 database

We can scrutinize the similarities and differences among species from the viewpoints of two major hypotheses: (a) *Pyrrhocoris apterus* and *P*. *tibialis* should be more similar to each other (at least in critical traits) than any of them to *Scantius aegyptius* since they are more closely related as indicated by their generic classification (*Pyrrhocoris* x *Scantius*), (b) *P*. *apterus* and *S*. *aegyptius* should be more similar since they are Müllerian (or quasi-Batesian; the term referring to mimetic relations between unequally defended species [56–58]) mimics sharing the same conspicuous aposematic coloration and occurring together at the same localities, habitats and host plants.

It can be seen in Table 5 that 24 compounds (24 in males, 24 in females) were identified in the defensive secretions of *Pyrrhocoris apterus*, namely 6 hydrocarbons (mainly dodecane, tridecane and pentadecane), 3 alcohols, 7 aldehydes (especially (*E*)-2-hexenal, (*E*,*E*)-2,4-hexadienal, nonanal, (*E*)-2-octenal and decanal), 5 esters (mainly methyl 2-hydroxy-3-methyl pentanoate) and 3 organic acids and in addition 1-methoxyoctane. With the exception of 2-ethylhexanoic acid and 2-propyl-1-pentanol these compounds were found in the both sexes. In comparison with the results obtained by Farine et al. [20] the profiles of the main compounds were similar and 10 from 35 compounds found by Farine matched (especially (*E*)-2-octenal, (E)-2-hexenal, tridecane and methyl 2-hydroxy-3-methyl pentanoate). The prevalent less polar or non-polar compounds identified in the secretions by Farine may correspond to using non-polar solvent (pentane) for gland extraction. Moreover, the samples extracted with pentane were concentrated under nitrogen flow and therefore, some volatile compounds could be lost.

Table 5 shows that the defensive secretions of *Pyrrhocoris tibialis* (altogether 41 compounds; 36 in males, 41 in females) were found to contain 11 hydrocarbons, 9 alcohols (mainly 1-dodecanol, (*E*)-2-hexen-1-ol and (*E*)-2-octen-1-ol), 8 aldehydes (especially (*E*)-2-octenal, nonanal and (*E*)-2-hexenal), 2 ketones and 8 esters of carboxylic acids. The secretions also contained limonene, probably derived from the seeds with which the insects were fed, and also 3-methylbutanoic acid and smaller amounts of 1-ethoxynaphthalene.

Composition of defensive secretions of *Scantius aegyptius* (altogether 38 compounds; 37 in males, 38 in females) contained 10 hydrocarbons, 10 alcohols (mainly 2-cyclohexen-1-ol, cyclopentanemethanol and 1-dodecanol), 3 aldehydes (nonanal, decenal and α-hexylcinnamaldehyde), 2 ketones and 11 esters of carboxylic acid. The secretions also contained limonene and smaller amounts of 1-ethoxynaphtalene.

Most of the compounds found in the secretions are highly volatile substances with low molecular weights and we may assume that most of them have either defensive function or act as intraspecific chemical signals. Saturated short-chain aldehydes are very effective chemical irritants, hydrocarbons can facilitate penetration of reactive aldehydes through the cuticle of arthropod predators and some esters can also assist in wetting the cuticle of arthropod predators [20]. The secretions of all the studied species also contained a large number of minority branched hydrocarbons (especially methylated) which were not included in Table 5 because the determination of methyl group position is difficult without reference standards.

**Fig 2:**
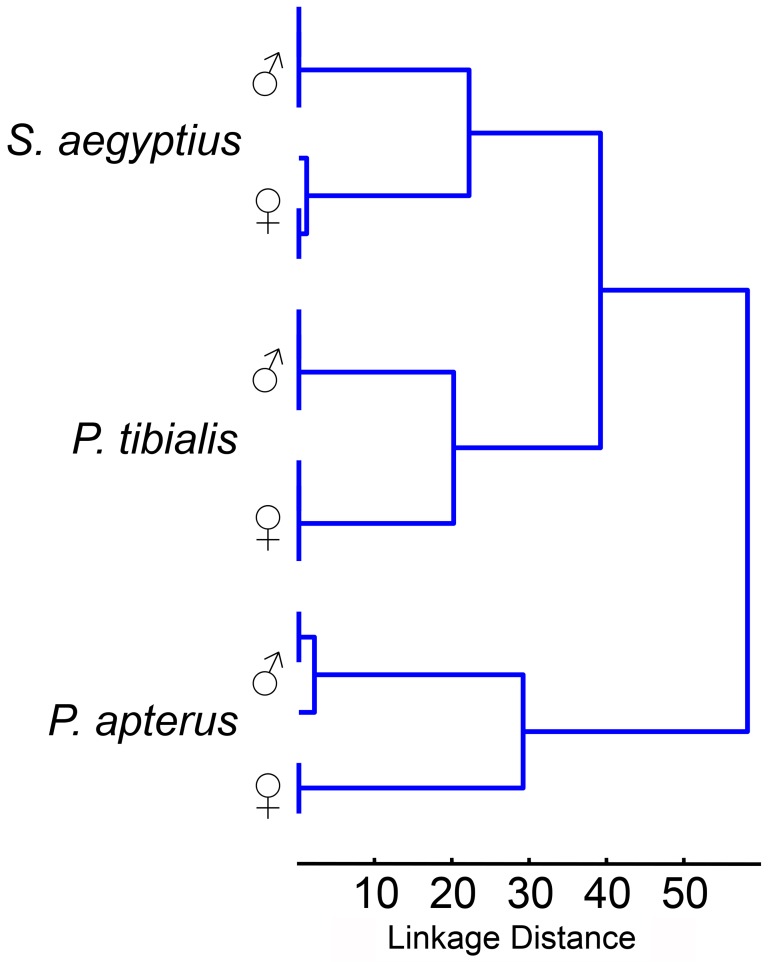
Dendrogram depicting the qualitative similarities in chemical profiles among the analysed sampled. The clustering was based on distance matrix, in which the distance between each pair of samples was expressed as a number of compounds exclusively present in one of the samples and absent in the other. All 135 detected compounds were considered, single linkage clustering algorithm was used for tree construction (for details, see Section *Comparison of secretions between true-bug species*).

Defensive secretions of *S*. *aegyptius* and *P*. *tibialis* match in 24 compounds, whereas defensive secretions of *P*. *apterus* and *S*. *aegyptius* match in 9, and *P*. *apterus* and *P*. *tibialis* in 14 compounds (all unisexual occurrences counted as presence in a species). A cluster tree based on qualitative phenetic similarities of the chemical profiles of individual pyrrhocorid species (with sexes considered as separate entities) is shown in Fig 2.

It seems that interspecific differences and similarities reflect neither phylogenetic relationships between the species nor mimicry in colour pattern between *P*. *apterus* and *S*. *aegyptius*. The absence of several compounds in *P*. *apterus* may reflect its greagarious life style not requiring long-distance intraspecific communication. Some of the similarities and differences may also result from different natural host plants whose seeds were used for feeding the cultured populations.

In the present samples we find several patterns of shared similarity of chemicals. Those present in all the three species are probably conservative plesimorphic characters of the clade containing genera *Pyrrhocori*s (with *P*. *apterus* and *P*. *tibialis*) and *Scantius* (with *S*. *aegyptius)*. Their number is high in hydrocarbons (6 compounds out of 12), but very low in alcohols (1 out of 17), aldehydes (2 out of 9) and nil in esters (0 out of 18), ketons (0 out of 4) and “others” (0 out of 6). This suggests that occurrence of the individual compound of alcohols, aldehydes and esters is mostly species-specific, while the few common ones of these groups (1-dodecanol, nonanal and decanal) are probably essential for functioning of the whole system.

The shared presence of a compound in two species is complementary to absence of this particular compound (or its replacement or transformation) in the third species. This happened in 3 hydrocarbons, 3 alcohols and 6 esters shared by *P*. *tibialis* and *S*. *aegyptius*, and 4 aldehydes shared by *P*. *apterus* and *P*. *tibialis*. Strangely, no compounds are shared exclusively by *P*. *apterus* and *S*. *aegyptius*. Of the complementary absences, there are 3 hydrocarbons, 3 alcohols and 6 esters missing in *P*. *apterus*, 4 aldehydes in *S*. *aegyptius*, but no unique absence was found in *P*. *tibialis*.

The secretion of the two *Pyrrhocoris* species contained greater number of aldehydes than the secretion of *Scantius aegyptius* (Table 5, 6–8 versus 3). Since the aldehydes are known to have antipredatory defensive function [6, 59, 60], this difference corresponds with the difference in palatability of *P*. *apterus* and *S*. *aegyptius* for avian predators (A. Exnerova et al., unpublished), where *S*. *aegyptius* appears to be considerably less well defended of the two species and may be a quasi-Batesian mimic of *P*. *apterus*. Moreover, most of the aldehydes are shared by both *Pyrrhocoris* species, with (*E*)-2-hexenal being dominant. Thus the composition of aldehydes appears rather to follow the phylogenetic relationships between the species than be a result of mimetic convergence.

The composition of hydrocarbons was mostly similar in all the three species, with only some of them missing in *P*. *apterus*, which is likely to represent a derived situation, convergence between two non-congeneric species (*P*. *tibialis* and *S*. *aegyptius*) is less likely. In all the three species, one of the dominant hydrocarbons is tridecane, known for its antipredatory function against arthropod predators, where it facilitates penetration of aldehydes through the cuticle [61].

Interspecific differences in the composition of alcohols and esters are difficult to interpret, because of their less known and possibly multiple and context-dependent functions [6, 60, 62]. High proportion of both alcohols and esters is species-specific, which indicates their interspecific signaling function [60, 62–64]. The strikingly smaller number of different alcohols and esters in *P*. *apterus* may be connected with its gregarious lifestyle with smaller need for long-distance chemical communication.

Limonene, which is like other terpenoids obtained by the bugs from their host plants, was present only in *S*. *aegyptius* and *P*. *tibialis*. Its absence in *P*. *apterus* may indicate its unavailability in linden seeds in contrast to those of *Alcea* and *Hibiscus*. Along with other terpenoids, limonene plays role in a chemical defense of the bugs, as it is serves as an ant alarm pheromone [65].

**Fig 3:**
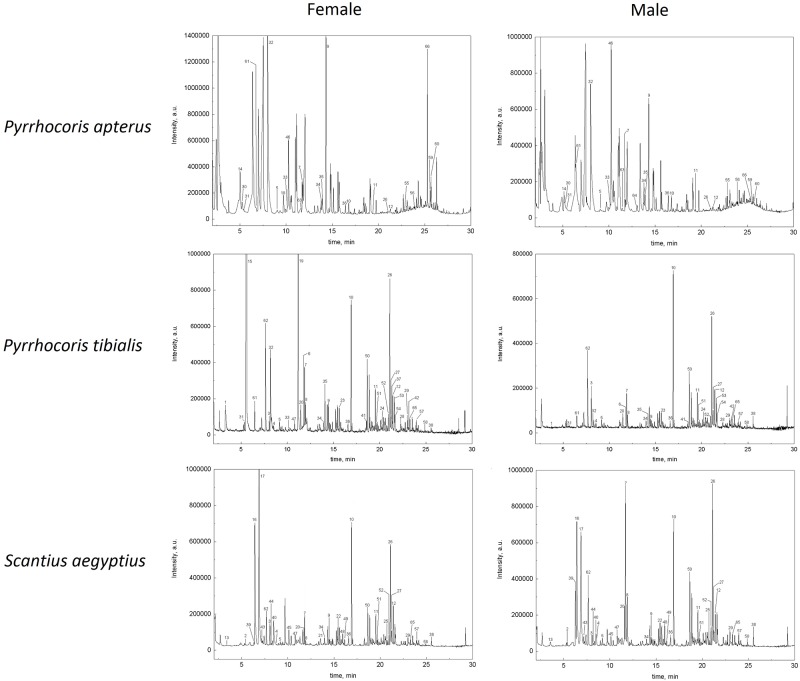
SPME-GC-MS analysis of the all studied true bugs. Sampling the secretion by compression with the plunger of a syringe; SPME sorption on a DVB/CAR/PDMS fiber; details of the sampling conditions given in Table 4; numbering of the peaks corresponds to Table 5.

Chromatograms obtained for the secretions produced by the males and females of all the studied true bugs are given in Fig 3.

The chromatograms obtained for male and female of the individual species differ slightly. Whereas three dominant peaks corresponding to tridecane (9), (*E*)-2-hexenal (32) and methyl 2-hydroxy-3-methyl pentanoate (46) are present in the male, as well as in the female chromatograms of *P*. *apterus*, 2-propyl-1-pentanol (18) is only found in females. The chromatogram of *P*. *tibialis* female is enriched by five additional compounds, especially by (*E*)-2-hexen-1-ol (15) and (*E*)-2-octen-1-ol (19), whereas male and female chromatograms of *S*. *aegyptus* differ especially in the relative abundance of dodecane (7), 2-cyclohexen-1-ol (17) and cyclopentanone (39). Generally, the secretions of females of all the three species contained larger number of alcohols than the secretion of conspecific males (Table 5). This may suggest the epigamic pheromonal function of the particular alcohols [62]. On the other hand, there was minimum of sexual dimorphism in composition of aldehydes, supporting their interspecific function in an antipredatory defense [6, 59, 60], and in composition of esters, which were reported as non-epigamic intraspecific attractants and alarm pheromones [60, 63, 64].

In no true bug species the exact function of numerous infochemicals of the secretion is known—in antipredatory context it can range from signaling to irritation up to being lethally toxic [66]. In addition to metathoracic glands representing the major source of defense infochemicals in adults, also other specialized glands and sequestered noxious plant compounds may play role in defense [7, 59]. It should be born in mind that also non-defensive infochemicals may be detected in the adults of true bugs (e.g. pheromones produced in dorsoabdominal glands persisting in adults from larval stage though with an altered function and no more secreting defense substances). We should emphasize that a similar extraction “in vivo” as having been used presently is also applicable to other arthropods (e.g., spiders, millipedes, a variety of insects in addition to true bugs), and may be used for all kinds of a rich array of exocrinous, supra-integumentally released, and often extremely complex defensive secretions.

## Conclusions

A set of non-lethal methods has been developed for SPME sampling of the volatile secretions of three species of true bugs (Insecta: Hemiptera: Heteroptera: Pyrrhocoridae) as a part of their defensive mechanisms. Particular attention was paid to optimization of the input parameters, including the means of irritation, and the optimization was performed using multi-parameter response surface methodology. On the basis of the required information (maximum number of peaks, maximum sum of all the peaks), the best method seemed to be irritation of the bugs by compression with the plunger of a syringe and use of a composite DVB/CAR/PDMS fiber for SPME sampling. This method also simulates best the natural attack by a vertebrate predator. GC separation was performed using a column Rtx-200 with fluorinated stationary phase providing good selectivity even for substances with very different polarity scales.

The developed method was used for sampling of volatile defensive secretions of adult males and females of *Pyrrhocoris apterus*, *P*. *tibialis* and *Scantius aegyptius* (all Pyrrhocoridae). The method applied has the advantage that only several individuals of each sex are sufficient for the analysis and thus even rare and not easily available species can be analyzed. Moreover, the method is non-lethal for the insects, which may be later potentially used in further studies requiring live individuals. The outlined methods can be widely used in similar studies on other insects although the optimization procedure and fiber evaluation would be necessary to perform for any new species studied owing to large variation of chemicals potentially involved.

The chemical composition of secretion, particularly that of alcohols, aldehydes and esters, is species-specific in all the three pyrrhocorid species studied. The overall similarities in composition of secretion do not reflect the relationship of species or mimicry between *Pyrrhocoris apterus* and *Scantius aegyptius*. The similarity of secretions between the bug species is associated with their antipredatory strategies. For instance the small number of alcohols and esters in *Pyrrhocoris apterus* is probably associated with its high gregariousness while low numbers of aldehydes in *Scantius aegyptius* reflects its higher palatability. The sexual dimorphism in occurrence of particular compounds is largely limited to alcohols and suggests their epigamic intraspecific function.

## Supporting Information

S1 Table Ultrasonics. Experimental parameters, their levels and modeling experimental plan of the face centered central composite design for sampling secretion using ultrasonics.
(DOCX)

S2 Table Shaker. Experimental parameters, their levels and modeling experimental plan of the face centered central composite design for sampling secretion using shaker.
(DOCX)

S3 Table Compression in the Plunger of a Syringe. Experimental parameters, their levels and modeling experimental plan of the face centered central composite design for sampling secretion using compression with the plunger of a syringe.
(DOCX)
