# Supplementary material for: Comparative Analysis of Volatile Defensive Secretions of Three Species of Pyrrhocoridae (Insecta: Heteroptera) by Gas Chromatography-Mass Spectrometric Method
Source: PLoS One. 2016 Dec 20;11(12):e0168827. doi: 10.1371/journal.pone.0168827 (PMC5173376; doi:10.1371/journal.pone.0168827)
Supplement: S1 Table — Experimental parameters, their levels and modeling experimental plan of the face centered central composite design for sampling secretion using ultrasonics. (DOCX) [file pone.0168827.s001.docx]

**S1 Table.**

|  | type of fiber | temperature of SPME sorption (°C) | SPME sampling time (min) | irritation time (min) | irritation temperature (°C) | intensity of the ultrasonics (%) | sum of peaks | sum of absolute peak areas |
| --- | --- | --- | --- | --- | --- | --- | --- | --- |
| 1 | DVB/CAR/PDMS | 25 | 30 | 1 | 25 | 100 | 2 | 130787 |
| 2 | PA | 32.5 | 60 | 3 | 32.5 | 65 | 3 | 153977 |
| 3 | PA | 32.5 | 60 | 3 | 32.5 | 65 | 1 | 71431 |
| 4 | PDMS | 25 | 30 | 5 | 40 | 30 | 1 | 42694 |
| 5 | PDMS | 40 | 90 | 1 | 25 | 30 | 6 | 434014 |
| 6 | DVB/CAR/PDMS | 40 | 90 | 5 | 40 | 100 | 13 | 6141928 |
| 7 | PA | 32.5 | 60 | 3 | 32.5 | 65 | 1 | 20366 |
| 8 | PA | 32.5 | 60 | 3 | 32.5 | 65 | 1 | 20366 |
| 9 | DVB/CAR/PDMS | 40 | 90 | 1 | 25 | 100 | 28 | 59037093 |
| 10 | DVB/CAR/PDMS | 25 | 30 | 5 | 40 | 100 | 3 | 116543 |
| 11 | PDMS | 40 | 90 | 5 | 40 | 30 | 24 | 1639520 |
| 12 | PDMS | 25 | 30 | 1 | 25 | 30 | 0 | 0 |
| 13 | PA | 32.5 | 60 | 3 | 32.5 | 65 | 0 | 0 |
| 14 | PA | 32.5 | 60 | 3 | 32.5 | 65 | 0 | 0 |
| 15 | PA | 32.5 | 60 | 3 | 32.5 | 65 | 0 | 0 |
| 16 | PA | 32.5 | 60 | 3 | 32.5 | 65 | 4 | 100536 |
| 17 | PA | 32.5 | 60 | 3 | 32.5 | 65 | 4 | 100536 |
| 18 | DVB/CAR/PDMS | 25 | 90 | 1 | 40 | 30 | 3 | 131943 |
| 19 | DVB/CAR/PDMS | 40 | 30 | 5 | 25 | 30 | 0 | 0 |
| 20 | PDMS | 40 | 30 | 1 | 40 | 100 | 27 | 1503406 |
| 21 | PDMS | 25 | 90 | 5 | 25 | 100 | 7 | 250935 |
| 22 | DVB/CAR/PDMS | 25 | 90 | 5 | 25 | 30 | 1 | 21315 |
|  | type of fiber | temperature of SPME sorption (°C) | SPME sampling time (min) | irritation time (min) | irritation temperature (°C) | intensity of the ultrasonics (%) | sum of peaks | sum of absolute peak areas |
| 23 | DVB/CAR/PDMS | 40 | 30 | 1 | 40 | 30 | 2 | 428591 |
| 24 | PA | 32.5 | 60 | 3 | 32.5 | 65 | 4 | 100536 |
| 25 | PDMS | 40 | 30 | 5 | 25 | 100 | 7 | 383588 |
| 26 | PDMS | 25 | 90 | 1 | 40 | 100 | 6 | 294292 |
| 27 | PA | 32.5 | 60 | 3 | 32.5 | 65 | 0 | 0 |
| 28 | PA | 32.5 | 60 | 3 | 32.5 | 65 | 0 | 0 |
| 29 | PDMS | 32.5 | 60 | 3 | 32.5 | 65 | 6 | 339090 |
| 30 | DVB/CAR/PDMS | 32.5 | 60 | 3 | 32.5 | 65 | 4 | 177440 |
| 31 | PA | 32.5 | 60 | 3 | 25 | 65 | 2 | 331136 |
| 32 | PA | 32.5 | 60 | 3 | 40 | 65 | 2 | 81910 |
| 33 | PA | 32.5 | 60 | 1 | 32.5 | 65 | 0 | 0 |
| 34 | PA | 32.5 | 60 | 5 | 32.5 | 65 | 2 | 228671 |
| 35 | PA | 32.5 | 30 | 3 | 32.5 | 65 | 0 | 0 |
| 36 | PA | 32.5 | 90 | 3 | 32.5 | 65 | 2 | 124405 |
| 37 | PA | 32.5 | 60 | 3 | 32.5 | 30 | 3 | 162940 |
| 38 | PA | 32.5 | 60 | 3 | 32.5 | 100 | 3 | 104338 |
| 39 | PA | 25 | 60 | 3 | 32.5 | 65 | 2 | 88649 |
| 40 | PA | 40 | 60 | 3 | 32.5 | 65 | 4 | 598018 |

Experimental parameters, their levels and modeling experimental plan of the face centered central composite design for sampling secretion using ultrasonics.
