# Supplementary material for: Comparative Analysis of Volatile Defensive Secretions of Three Species of Pyrrhocoridae (Insecta: Heteroptera) by Gas Chromatography-Mass Spectrometric Method
Source: PLoS One. 2016 Dec 20;11(12):e0168827. doi: 10.1371/journal.pone.0168827 (PMC5173376; doi:10.1371/journal.pone.0168827)
Supplement: S2 Table — Experimental parameters, their levels and modeling experimental plan of the face centered central composite design for sampling secretion using shaker. (DOCX) [file pone.0168827.s002.docx]

**S2 Table.**

|  | type of fiber | temperature of SPME sorption (°C) | SPME sampling time (min) | irritation time (min) | irritation temperature (°C) | rate of shaking (rpm) | sum of peaks | sum of absolute peak areas |
| --- | --- | --- | --- | --- | --- | --- | --- | --- |
| 1 | DVB/CAR/PDMS | 40 | 30 | 5 | 25 | 300 | 17 | 16388957 |
| 2 | PDMS | 25 | 90 | 5 | 25 | 1200 | 0 | 0 |
| 3 | PDMS | 40 | 90 | 1 | 40 | 300 | 6 | 288879 |
| 4 | PA | 32.5 | 60 | 3 | 32.5 | 750 | 1 | 25695 |
| 5 | DVB/CAR/PDMS | 25 | 30 | 1 | 40 | 1200 | 1 | 66049 |
| 6 | DVB/CAR/PDMS | 25 | 30 | 5 | 25 | 1200 | 0 | 0 |
| 7 | PDMS | 25 | 90 | 1 | 40 | 1200 | 3 | 91997 |
| 8 | PA | 32.5 | 60 | 3 | 32.5 | 750 | 0 | 0 |
| 9 | DVB/CAR/PDMS | 40 | 30 | 1 | 40 | 300 | 8 | 1524679 |
| 10 | PDMS | 40 | 90 | 5 | 25 | 300 | 0 | 0 |
| 11 | DVB/CAR/PDMS | 25 | 90 | 5 | 40 | 300 | 3 | 164854 |
| 12 | PDMS | 40 | 30 | 5 | 40 | 1200 | 0 | 0 |
| 13 | PA | 32.5 | 60 | 3 | 32.5 | 750 | 2 | 45404 |
| 14 | PDMS | 25 | 30 | 1 | 25 | 300 | 0 | 0 |
| 15 | DVB/CAR/PDMS | 40 | 90 | 1 | 25 | 1200 | 6 | 523762 |
| 16 | PDMS | 25 | 30 | 5 | 40 | 300 | 0 | 0 |
| 17 | PA | 32.5 | 60 | 3 | 32.5 | 750 | 0 | 0 |
| 18 | DVB/CAR/PDMS | 40 | 90 | 5 | 40 | 1200 | 4 | 1064752 |
| 19 | PDMS | 40 | 30 | 1 | 25 | 1200 | 0 | 0 |
| 20 | DVB/CAR/PDMS | 25 | 90 | 1 | 25 | 300 | 0 | 0 |
| 21 | PDMS | 32.5 | 60 | 3 | 32.5 | 750 | 0 | 0 |
| 22 | PDMS | 32.5 | 60 | 3 | 32.5 | 750 | 1 | 55664 |
|  | type of fiber | temperature of SPME sorption (°C) | SPME sampling time (min) | irritation time (min) | irritation temperature (°C) | rate of shaking (rpm) | sum of peaks | sum of absolute peak areas |
| 23 | PA | 32.5 | 60 | 3 | 25 | 750 | 1 | 23406 |
| 24 | PA | 32.5 | 60 | 3 | 40 | 750 | 0 | 0 |
| 25 | PA | 32.5 | 60 | 1 | 32.5 | 750 | 1 | 49336 |
| 26 | PA | 32.5 | 60 | 5 | 32.5 | 750 | 0 | 0 |
| 27 | PA | 32.5 | 60 | 3 | 32.5 | 300 | 0 | 0 |
| 28 | PA | 32.5 | 60 | 3 | 32.5 | 1200 | 0 | 0 |
| 29 | PA | 32.5 | 30 | 3 | 32.5 | 750 | 0 | 0 |
| 30 | PA | 32.5 | 90 | 3 | 32.5 | 750 | 1 | 38975 |
| 31 | PA | 25 | 60 | 3 | 32.5 | 750 | 1 | 26865 |
| 32 | PA | 40 | 60 | 3 | 32.5 | 750 | 2 | 125613 |

Experimental parameters, their levels and modeling experimental plan of the face centered central composite design for sampling secretion using shaker.
