# Supplementary material for: Comparative Analysis of Volatile Defensive Secretions of Three Species of Pyrrhocoridae (Insecta: Heteroptera) by Gas Chromatography-Mass Spectrometric Method
Source: PLoS One. 2016 Dec 20;11(12):e0168827. doi: 10.1371/journal.pone.0168827 (PMC5173376; doi:10.1371/journal.pone.0168827)
Supplement: S3 Table — Experimental parameters, their levels and modeling experimental plan of the face centered central composite design for sampling secretion using compression with the plunger of a syringe. (DOCX) [file pone.0168827.s003.docx]

**S3 Table.**

|  |  |  |  |  | SPME fiber: PDMS | | SPME fiber: PA | | SPME fiber: DVB/CAR/PDMS | |
| --- | --- | --- | --- | --- | --- | --- | --- | --- | --- | --- |
|  | temperature of SPME sorption (°C) | time of SPME sorption (min) | temperature prior to compression (°C) | tempering time prior to compression (min) | sum of peaks | sum of absolute peak areas | sum of peaks | sum of absolute peak areas | sum of peaks | sum of absolute peak areas |
| 1 | 40 | 30 | 25 | 5 | 9 | 301277 | 2 | 153218 | 23 | 2357339 |
| 2 | 25 | 90 | 40 | 1 | 2 | 74631 | 2 | 176044 | 23 | 47198219 |
| 3 | 40 | 30 | 40 | 1 | 3 | 78343 | 2 | 64363 | 26 | 2597821 |
| 4 | 25 | 90 | 25 | 5 | 7 | 950464 | 2 | 272356 | 28 | 6740215 |
| 5 | 40 | 90 | 25 | 1 | 3 | 100427 | 0 | 0 | 49 | 5754942 |
| 6 | 40 | 90 | 40 | 5 | 0 | 0 | 1 | 95558 | 63 | 22101121 |
| 7 | 25 | 30 | 25 | 1 | 10 | 1114880 | 0 | 0 | 25 | 13341150 |
| 8 | 25 | 30 | 40 | 5 | 0 | 0 | 0 | 0 | 10 | 540351 |
| 9 | 32.5 | 60 | 25 | 3 | 0 | 0 | 2 | 125803 | 26 | 2923929 |
| 10 | 32.5 | 60 | 40 | 3 | 1 | 56455 | 4 | 223415 | 20 | 5533938 |
| 11 | 32.5 | 60 | 32.5 | 1 | 1 | 54937 | 1 | 322002 | 32 | 31266771 |
| 12 | 32.5 | 60 | 32.5 | 5 | 1 | 29111 | 26 | 25900711 | 23 | 7945168 |
| 13 | 32.5 | 30 | 32.5 | 3 | 4 | 273405 | 2 | 68057 | 20 | 3093574 |
| 14 | 32.5 | 90 | 32.5 | 3 | 13 | 1894277 | 1 | 32798 | 37 | 9036632 |
| 15 | 25 | 60 | 32.5 | 3 | 2 | 90775 | 3 | 94260 | 14 | 2253231 |
| 16 | 40 | 60 | 32.5 | 3 | 2 | 54996 | 2 | 66299 | 25 | 13559193 |

Experimental parameters, their levels and modeling experimental plan of the face centered central composite design for sampling secretion using compression with the plunger of a syringe for three different SPME fibers.
